# Exploration of the methods of establishing the minimum clinical important difference based on anchor and its application in the quality of life measurement scale QLICP-ES (V2.0) for esophageal cancer

**DOI:** 10.1186/s12955-021-01808-7

**Published:** 2021-07-02

**Authors:** Dandan Ren, Ting Wu, Chonghua Wan, Gaofeng Li, Yanbo Qi, Yujing Fang, Jiudi Zhong

**Affiliations:** 1 School of Humanities and Management, Research Center for Quality of Life and Applied Psychology, Guangdong Medical University, Dongguan, 523808 China; 2 The Third Affiliated Hospital, Kunming Medical University (Yunnan Tumor Hospital), Kunming, 650106 China; 3 The Center for Response and Management of Emergence Public Health Event, the Center for Disease Control and Prevention of Yunnan Province, Kunming, 650022 China; 4 Department of Thoracic Surgery, State Key Laboratory of Oncology in South China, Sun Yat-Sen University Cancer Center, Collaborative Innovation Center of Cancer Medicine, Guangzhou, 510060 China

**Keywords:** Quality of life, Esophageal cancer, The minimum clinical important difference, Anchor-based method, ROC curve, Multiple linear regression model

## Abstract

**Background:**

The development of the minimum clinical important difference (MCID) can make it easier for researchers or doctors to judge the significance of research results and the effect of intervention measures, and improve the evaluation system of efficacy. This paper is aimed to calculate the MCID based on anchor and to develop MCID for esophageal cancer scale (QLICP-ES).

**Methods:**

The item Q29 (How do you evaluate your overall health in the past week with 7 grades answers from 1 very poor to 7 excellent)of EORTC QLQ-C30 was used as the subjective anchor to calculate the score difference between each domain at discharge and admission. MCID was established according to two standards, "one grade difference"(A) and "at least one grade difference"(B), and developed by three methods: anchor-based method, ROC curve method and multiple linear regression models. In terms of anchor-based method, the mean of the absolute value of the difference before and after treatments is MCID. The point with the best sensitivity and specificity-Yorden index at the ROC curve is MCID for ROC curve method. In contrast, the predicted mean value based on a multiple linear regression model and the parameters of each factor is MCID.

**Results:**

Most of the correlation coefficients of Q29 and various domains of the QLICP-ES were higher than 0.30. The rank of MCID values determined by different methods and standards were as follows: standard B > standard A, anchor-based method > ROC curve method > multiple linear regression models. The recommended MCID values of physical domain, psychological domain, social domain, common symptom and side-effects domain, the specific domain and the overall of the QLICP-ES were 7.8, 9.7, 4.7, 3.6, 4.3, 2.3 and 2.9, respectively.

**Conclusion:**

Different methods have their own advantages and disadvantages, and also different definitions and standards can be adopted according to research purposes and methods. A lot of different MCID values were presented in this paper so that it can be easy and convenient to select by users.

## Background

Esophageal cancer is the second most common solid intrathoracic malignancy behind lung cancer and the sixth leading cause of cancer death in the world [1, 2]. The incidence and death of esophageal cancer in China account for about 50% of the world's cases [3]. The proportion of male and female patients dying is about 2:1, and most of them are over 40 years old [4]. The typical symptom of esophageal cancer is progressive dysphagia, due to various treatments only to prolong survival, how to improve the quality of life (QOL) of patients becomes the concerning in field of medicine. Consequently, a lot of measuring instruments such as QOL/PRO and mental health scales have been developed and are widely used in clinical practices and researches.

However, the interpretation of the scores of the scale is usually judged by the *P* value, more and more scholars have realized that it is not reasonable and scientific to judge the curative effect only according to the different scale scores before and after the treatments (hypothesis test *P* values). In fact, the *P* value will become statistically significant as the samples being big enough, which does not mean having clinical significance [5]. Therefore, a key question in the application of the scale is how much its score must change to be clinically meaningful, i.e. the minimum clinical important difference (MCID).

There are different names and meanings for MCID: minimal important difference (MID) [6], minimal clinically important change (MCIC) [7], the smallest detectable difference (SDD), minimal detectable change (MDC) [8], sufficient important difference (SID) [9], etc. Also there are several calculation standards with its name and standard having not been completely unified [10–13]: deterioration, a little deterioration, no change, a little improvement, improvement, etc.

There are several methods for the formulation of MCID, among which there are two traditional methods including anchor-based methods and distribution-based methods. Although the traditional methods have their advantages, their shortcomings and limitations are gradually exposed for there is no unified standard. The major disadvantage of all methods using the distribution-based approach is that they do not, in themselves, provide a good indication of the importance of the observed change [14]. Anchor-based method is a traditional and widely used method, which can verify the significance of changes through external indicators based on patients' subjective feelings [15]. It also has many problems, such as it is difficult for determination of effects standards and suitable anchor, selection of mean and median according to scores distributions, the appropriateness of using clinical objective indicators as objective anchors, and so on [16, 17].

In recent years, some new methods have been proposed such as ROC curve method based on anchor and multiple linear regression model [18, 19]. The ROC curve method integrates the anchor-based method and the distribution-based method. The ROC approach integrates type-one and type-two errors and lie within reasonable limits, and it is suitable for data that is not normally distributed [20, 21]. But the MIC_ROC_ is very sensitive to random sampling variation, especially in relatively small samples. It is difficult to identify the cut-off point with the best sensitivity and specificity at one glance in the ROC curve. Multiple linear regression model as an extension of the average change method, opens a new field of vision, which can be extended by covariables that may causes confusion and may not be equally distributed between the transition categories, for example, sex, age [22].

Therefore, it is important and urgent need to deal with the existed problems of the classical anchor-based methods, and to explore the appropriateness of new methods. This paper is aimed to discuss in detail how to apply the classical anchor-based methods, especially the new methods in recent years, to formulate MCID for the Quality of Life Measurement Scale QLICP-ES (V2.0) for Esophageal Cancer, and to compare the values of MCID under different methods and standards.

## Methods

### Survey methods

*Survey object* Patients with esophageal cancer treated at Yunnan Cancer Hospital and Sun Yat-sen University Cancer Prevention Center. Inclusion criteria: (1) patients with a clear diagnosis of esophageal cancer; able to fill out the questionnaire by themselves; (2) volunteer to participate in the survey, without mental illness and consciousness disorder. Exclusion criteria: (1) cognitive dysfunction; (2) multiple metastases of malignant tumors; (2) refusal to participate in the study or those with a low degree of cooperation.

*Survey method* The investigators (doctors/ nurses/medical postgraduates) explained the aim of the test and the scales to the patients. The Participating patients were required to finish the informed consent form and the scales of QLICP-ES (V2.0) and Chinese version of EORTC QLQ-C30 [23] independently on the day of admission to the hospital, and once again at the day before discharge.

Survey tool: The QLICP-ES (V2.0) is an esophageal cancer scale with Chinese cultural characteristics and background developed by our QOL team by modular approach, which has good reliability, validity and responsiveness [24]. The scale is composed of a general module QLICP-GM (V2.0) which can be used for all cancers and an esophageal cancer-specific module. Among them, the QLICP-GM (V2.0) includes 32 items grouped into four domains: physical function (8 items), psychological function (9 items), social function (8 items), and common symptoms and side effects (7 items). The whole scale consists of 48 items with the specific module having 16 items, and the variables of basic demographic information such as gender, age, education level, family economic status, etc. being included as the first page of the scale.

*Scoring method* The raw scores of items, domains and overall scale were calculated for the QLICP-ES according to the scoring guide, with each domain score being obtained by adding its own item score together and the overall score being the sum of five domains score. And all domains and the overall scores were linearly converted to a 0–100 scale standardized scores.

Treatment of missing values: If the missing rate of the scale is > 5%, it is considered as an invalid scale. If the missing rate of the scale is < 5%, the score of the missing item will be replaced with the median score of the item.

### Anchor-based method

The better method in formulation of MCID is anchor-based method, which was proposed by American scholar Lydick et al. [25] in 1993. Its principle is to clarify the meaning of the rating change of the scale by examining the relationship between the scale and the score of another independent measurement tool or other indicators (i.e. anchors). First, an appropriate anchor was selected and the correlation coefficient between the anchor and the test scale was reported. Revicki et al. [12] believed that the correlation coefficient should be no less than 0.30–0.35. Second, MCID were calculated according to some standards defining the effects of treatments.

In this paper, the data were obtained from the self-matched experimental design, and the anchor-based method was mainly used to formulate the MCID of QLICP-ES. The 29th item of EORTC QLQ-C30, " Q29, how do you evaluate your overall health in the past week?", is used as the subjective anchor, with the answers including seven grades (from very poor to excellent). Pearson correlation analysis was used to calculate the correlation coefficients between Q29 and various domains.

The two effects standards of "one grade difference" (standard A) and "at least one grade difference" (standard B) were selected to develop MCID. The rationale of these classification standards was as following: if the score at the same anchor change one grade (including the rise and fall of a level) after the treatments/interventions, it implies that the patient has some important effects for its health status changed a grade. It is obvious that the later standard (at least one grade difference) imply greater health effects for smaller and larger changes. These two standards can be contrasted.

If $x_{0}$ represents respondents baseline score (on the day of admission), $x_{1}$ on behalf of the respondents rating score after interventions (the day before discharge), and then suitable patients according to two standards were selected and the difference d between two measuring points was computed. If the patient's anchor overall health improves, then *d* = $x_{1}$* – *$x_{0}$; if the patient's anchor overall health deteriorates, then *d* = $x_{0}$* – *$x_{1}$*.* The mean of the difference of all patients selected was as the MCID.

### ROC curve method

Both the anchor-based method and the distribution-based method have some disadvantages. Therefore, Crosby et al. [26] plead for a combination of anchor-based and distribution-based methods to take advantage of both an external criterion and a measure of variability. We call this method as ROC curve method for it is on the basis of ROC curve and anchor-based MIC distribution in nature [14].

First, using an anchor, the patients were divided into two groups: one grade difference/at least one grade difference, no change. Then the distribution of the change in scores on the health status instrument was plotted.

Second, the cut-off point for an MIC was chosen. Here two cut-off points were considered: the Receiver Operating Characteristic (ROC) cut-off point and the 95% limit cut-off point. The ROC cut-off point is the value for which the sum of percentages of false positive and false negative classifications ([1-sensitivity] + [1-specificity]) is smallest. The 95% limit cut-off point is based on the distribution of scores of these persons who are unchanged according to the anchor.

Next, using the 95% limit cut-off point, MIC for improvement is defined as the 95% upper limit of the distribution of scores of these persons who are unchanged according to the anchor [mean change + 1.645 SD_change_]. Note that the 95% limit cut-off point corresponds with 95% specificity on the ROC curve.

To determine the ROC cut-off point for each change in domain score, the sensitivity and specificity were calculated. To construct the ROC curve, the combination of sensitivity and 1-specificity for each change in domain scores was plotted. The MIC, defined as the optimal cut-off point, is found on the ROC curve at the point closest to the upper-left corner (i.e. where the sum of the percentages of misclassified patients is lowest).

### Multiple linear regression models

Angst et al. [22] in 2017 has put forward a MCID method by multiple linear regression models, with its advantage adjusting the potential confounding factors. The specific steps are as follows:

First, variables analyzed were determined by anchor options and also potential influence factors. The score change after treatments "$d_{{change}}$" was used as the dependent variable, and the classification group by anchor adopting two kinds of standards "one grade difference" and "at least one grade difference", and potential influence factors such as gender, age, level of education, family economic etc. were used as independent variables.

Second, multivariate linear regression models were built and the parameters and the predictive value of the mean were estimated by SPSS. The multivariate linear regression model for standard A was:

$$d_{{change}} = a_{0} a_{1} x_{1} a_{2} x_{2} \cdots a_{k} x_{k} .$$

where × 1: gender (male = 0, female = 1), × 2: age (≦ 60 = 0, > 60 = 1), × 3: education (primary = 1, middle = 2, high or technical secondary school = 3, junior college = 4, bachelor degree or above = 5), × 4: family economy (poor = 1, medium = 2, rich = 3), × 5: group (no change = 0, change a level = 1),  × 6: baseline score of domains. And ***a***_**1**_, ***a***_**2**_ … ***a***_**6**_ is the partial regression coefficients of $d_{{change}}$ with ***x***_**1**_, ***x***_**2**_, ***x***_**3**_, ***x***_**4**_, ***x***_**5**_, ***x***_**6**_, respectively.

The assignment of variables in Standard B is almost the same as standard A except for × 5: group (no change = 0, change by more than one level = 1).

After ***a***_**1**_, ***a***_**2**_…***a***_**6**_ estimated, the predictive value of the mean (i.e. MCID) can be calculated, and also its 95% confidence interval can be estimated.

Based on the empirical comparison of two different criteria and these anchor methods of ROC curve and multiple linear regression models, a reasonable calculation of MCID for the esophageal cancer scale (QLICP-ES) was carried out.

## Results

### Socio-demographic characteristics of the sample

The total sample included 232 cases of hospitalized patients with esophageal cancer aged 35 years to 82 years (median age = 60 years and mean age = 59.3 ± 8.9 years). 204 (87.9%) were male and 203 (87.5%) were of Han ethnicity. 139 (59.9%) have a fair perceived income. On education level, 81 cases (34.9%) finished primary school, while 129 (55.6%) completed high school, and 22 (9.5%) had a college or post-graduate degree.

220 patients (94.8%) completed the questionnaires at discharge (about four weeks follow-up) and the data were used for computing score change for each patient.

### Correlation coefficients of Q29 with domains of the QLICP-ES

According to Pearson correlation analysis, the correlation coefficients of Q29 and other domains were all higher than 0.30 showing a strong correlation (see Table 1 in detail), except for the correlation coefficients of Q29 and psychological function of 0.17, Revicki [12] et al. believed that the correlation coefficient should be no less than 0.30 ~ 0.35. In other words, Q29 could be used as a subjective anchor to calculate the MCID in all domains of the esophageal cancer scale.

**Table 1:** Correlation coefficients between Q29 and domains of the QLICP-ES

Item	PHD	PSD	SOD	SSD	SPD	CGM	TOT
Q29	0.69**	0.17*	0.32**	0.56**	0.68**	0.68**	0.75**

*PHD* physical domain, *PSD* psychological domain, *SOD* social domain, *SSD* common symptoms and side effect domain, *CGM* core/general module, *SPD *specific domain, *TO t*otal

*P < 0.05; **P < 0.01

### MCID by anchor-based method

When Q29 was taken as the subjective anchor, 55 patients had no change in anchor option after interventions, 102 patients had a change with difference of one grade, and 165 patients had a change with difference of at least one grade. As can be seen from Table 2, the MCID value of physical domain, psychological domain, social domain, common symptom and side-effects domain, the general module, the specific module and the overall scale under the standard A is 5.1, 4.4, 3.1, 6.7, 4.8, 8.5 and 6.0, respectively, and the MCID value of above domains and the overall scale under the standard B is 19.3, 4.2, 4.8, 7.7, 6.5, 9.5, and 7.5, respectively. The mean score changes are positive except of psychological function, and the MCID value obtained by the standard of "at least one grade difference" (standard B) is larger than that obtained by the standard of "one grade difference" (standard A). No matter standards, the MCID value of physiological function was larger than that of the other domains, ranging from 4 to 10.

**Table 2:** The MCID of QLICP-ES (V2.0) determined by anchor-based method (n_A_ = 102, n_B_ = 165)

Domain	Items	Standard A $\stackrel{-}{x}\pm s$	Standard B $\stackrel{-}{x}\pm s$	Standard A MCID	Standard B MCID
Physical domain (PHD)	8	15.1 ± 14.8	19.3 ± 16.1	15.1	19.3
Psychological domain (PSD)	9	− 4.4 ± 11.8	− 4.2 ± 12.4	4.4	4.2
Social domain (SOD)	8	3.1 ± 10.2	4.8 ± 11.2	3.1	4.8
Common symptoms and side effect domain (SSD)	7	6.7 ± 10.7	7.7 ± 11.3	6.7	7.7
Core/general module (CGM)	32	4.8 ± 6.5	6.5 ± 7.3	4.8	6.5
Specific domain (SPD)	16	8.5 ± 8.3	9.5 ± 9.2	8.5	9.5
Total (TOT)	48	6.0 ± 6.0	7.5 ± 6.8	6.0	7.5

### MCID by ROC curve method

**Fig. 1:**
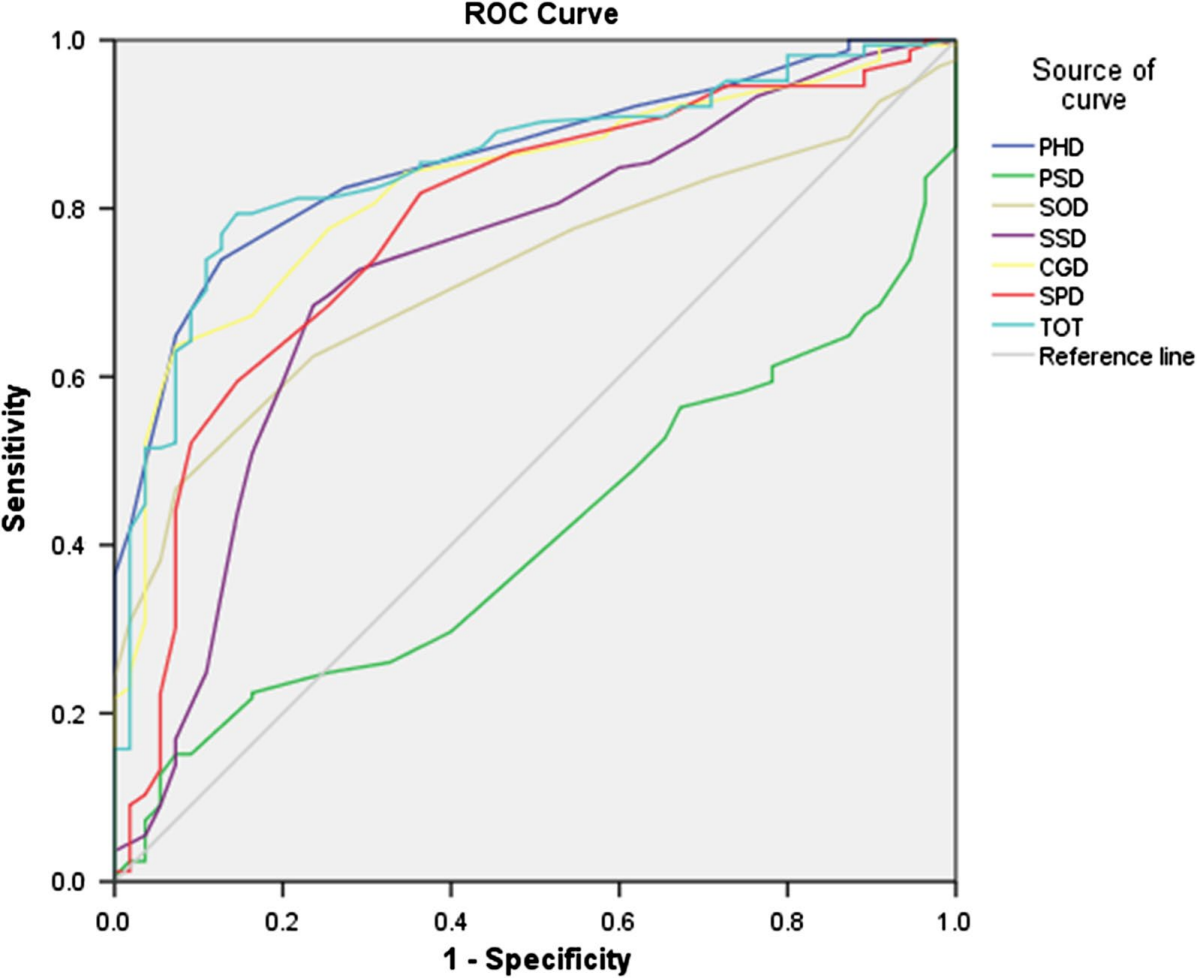
ROC curve of the group with no change and the group with change of at least one grade difference

**Fig. 2:**
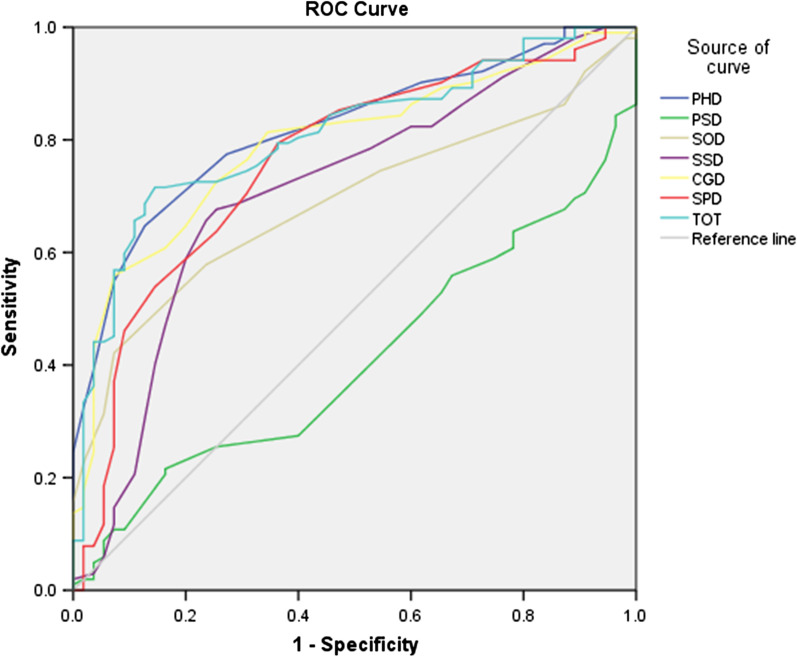
ROC curves of the group with no change and the group with change of one grade difference

The sample size of ROC curve method is different from that of the anchor-based method, which includes patients who had no change in anchor option after interventions. There are 157 patients under the A standard, and 220 patients under the B standard. The area under ROC curve (AUC) and MCID values of all domains of the QLICP-ES in each standard are shown in Table 3, Figs. 1 and 2, with Figs. 1 and 2 showing the ROC curves of each domain for standard A and standard B, respectively. As can be seen from Table 3, the MCID value of physical domain, psychological domain, social domain, common symptom and side-effects domain, the general module, the specific module and the overall scale under the standard A is 7.8, 9.7, 4.7, 3.6, 4.3, 2.3 and 2.9, respectively, and the MCID value of above domains and the overall scale under the standard B is 7.8, 5.6, 4.7, 3.6, 4.3, 7.0 and 2.9, respectively. The MCID values obtained by ROC curve method are consistent and relatively stable under the two standards, except for the psychological function and the specific module.

**Table 3:** The MCID of QLICP-ES (V2.0) determined by ROC curves (n_A_ = 157, n_B_ = 220)

Domain	Standard A AUC	Standard B AUC	Standard A MCID	Standard B MCID
Physical domain (PHD)	0.82	0.86	7.8	7.8
Psychological domain (PSD)	0.41	0.41	9.7	5.6
Social domain (SOD)	0.69	0.72	4.7	4.7
Common symptoms and side effect domain (SSD)	0.71	0.73	3.6	3.6
Core/general module (CGM)	0.79	0.83	4.3	4.3
Specific domain (SPD)	0.76	0.78	2.3	7.0
Total (TOT)	0.81	0.83	2.9	2.9

### MCID by multiple linear regression models

Table 4 presented the MCID values and regression models for different domains of the scale in standard A, as well as the *P* values and *R*^*2*^ of the models. As can be seen from Table 4, the MCID value of physical domain, psychological domain, social domain, common symptom and side-effects domain, the general module, the specific module and the overall scale under the standard A is 9.0, 3.0, 1.2, 4.0, 2.6, 5.9 and 3.7, respectively. Table 5 presented the MCID values and regression models for different domains of the scale in standard B, as well as the *P* values and *R*^*2*^ of the models. As can be seen from Table 5, the MCID value of above domains and the overall scale under the standard B is 13.9, 3.3, 3.0, 5.6, 4.5, 7.4 and 5.5, respectively. Also, it can be seen that the MCID value obtained by this method is similar to that of the traditional anchor method, and the MCID value obtained by standard B is larger than that obtained by standard A.

**Table 4:** The MCID of QLICP-ES (V2.0) determined by Multiple linear regression in standard A (n_A_ = 157)

_Domain_	_MCID_	_*P*_	*R*^*2*^	_Multiple linear regression model_
PHD	_9.0_	_< 0.001_	_0.27_	$d_{{change}}$ = − 15.46 − 2.81***x***_**1**_ + 1.01***x***_**2**_ − 1.21***x***_**3**_ + 4.05***x***_**4**_ + 16.37***x***_**5**_ + 0.13***x***_**6**_
PSD	_3.0_	_0.027_	_0.05_	$d_{{change}}$ = − 5.60 + 3.24***x***_**1**_ − 0.14***x***_**2**_ + 0.67***x***_**3**_ − 2.86***x***_**4**_ − 2.51***x***_**5**_ + 0.12***x***_**6**_
SOD	_1.2_	_< 0.001_	_0.12_	$d_{{change}}$ = 3.72 − 1.36***x***_**1**_ − 0.61***x***_**2**_ − 0.25***x***_**3**_ + 3.81***x***_**4**_ + 4.42***x***_**5**_ − 0.17***x***_**6**_
_SSD_	_4.0_	_< 0.001_	_0.22_	$d_{{change}}$ = 24.16 + 3.24***x***_**1**_ − 1.13***x***_**2**_ − 0.62***x***_**3**_ − 0.06***x***_**4**_ + 6.65***x***_**5**_ − 0.29***x***_**6**_
_CGM_	_2.6_	_< 0.001_	_0.22_	$d_{{change}}$ = 9.07 + 0.90***x***_**1**_ + 0.08***x***_**2**_ − 0.28***x***_**3**_ + 1.53***x***_**4**_ + 5.38***x***_**5**_ − 0.18***x***_**6**_
_SPD_	_5.9_	_<0.001_	_0.12_	$d_{{change}}$ = 3.13 + 1.26***x***_**1**_ + 0.50***x***_**2**_ − 0.35***x***_**3**_ + 0.90***x***_**4**_ + 7.23***x***_**5**_ − 0.04***x***_**6**_
TOT	_3.7_	_< 0.001_	_0.22_	$d_{{change}}$ = 3.86 + 0.93***x***_**1**_ + 0.27***x***_**2**_ − 0.33***x***_**3**_ + 1.13***x***_**4**_ + 6.23***x***_**5**_ − 0.08***x***_**6**_

$d_{{change}}$: the score difference between two measuring points, × 1: gender (male = 0, female = 1), × 2: age (≦ 60 = 0, > 60 = 1), × 3: education (Primary = 1, middle = 2, high or technical secondary school = 3, junior college = 4, bachelor degree or above = 5), × 4: family economy (poor = 1, medium = 2, rich = 3), × 5: group (no change = 0, Change a level = 1), × 6: field base points

*PHD* physical domain, *PSD* psychological domain, *SOD* social domain, *SSD* common symptoms and side effect domain, *CGM* core/general module, *SPD*specific domain, *TOT* total

**Table 5:** The MCID of QLICP-ES (V2.0) determined by Multiple linear regression in standard B (n_B_ = 220)

_Domain_	_MCID_	_*P*_	*R*^*2*^	_Multiple linear regression model_
PHD	_13.9_	_< 0.001_	_0.36_	$d_{{change}}$ = − 26.36 − 0.03***x***_**1**_ − 0.73***x***_**2**_ − 0.38***x***_**3**_ + 1.62***x***_**4**_ + 19.76***x***_**5**_ + 0.35***x***_**6**_
PSD	_3.3_	_0.002_	_0.07_	$d_{{change}}$ = − 13.37 + 0.08***x***_**1**_ + 0.22***x***_**2**_ + 0.63***x***_**3**_ − 2.10***x***_**4**_ − 1.72***x***_**5**_ + 0.21***x***_**6**_
SOD	_3.0_	_0.002_	_0.07_	$d_{{change}}$ = − 7.83 + 0.71***x***_**1**_ − 0.66***x***_**2**_- − 0.46***x***_**3**_ + 1.45***x***_**4**_ + 6.53***x***_**5**_ + 0.06***x***_**6**_
_SSD_	_5.6_	_< 0.001_	_0.13_	$d_{{change}}$ = 15.90 + 1.39***x***_**1**_ − 0.48***x***_**2**_ + 0.20***x***_**3**_ − 0.79***x***_**4**_ + 8.57***x***_**5**_ + 0.20***x***_**6**_
_CGM_	_4.5_	_< 0.001_	_0.18_	$d_{{change}}$ = 1.53 + 1.07***x***_**1**_ − 0.63***x***_**2**_ + 0.08***x***_**3**_ + 0.50***x***_**4**_ + 7.72***x***_**5**_ − 0.05***x***_**6**_
_SPD_	_7.4_	_< 0.001_	_0.12_	$d_{{change}}$ = − 2.42 + 0.79***x***_**1**_ + 0.18***x***_**2**_ − 0.26***x***_**3**_ + 0.43***x***_**4**_ + 7.92***x***_**5**_ + 0.04***x***_**6**_
TOT	_5.5_	_< 0.001_	_0.21_	$d_{{change}}$ = − 1.25 + 0.98***x***_**1**_ − 0.33***x***_**2**_ − 0.05***x***_**3**_ + 0.43***x***_**4**_ + 7.91***x***_**5**_ + 0.01***x***_**6**_

$d_{{change}}$: the score difference between two measuring points, × 1: gender (male = 0, female = 1), × 2: age (≦ 60 = 0, > 60 = 1), × 3: education (Primary = 1, middle = 2, high or technical secondary school = 3, junior college = 4, bachelor degree or above = 5), × 4: family economy (poor = 1, medium = 2, rich = 3), × 5: group (no change = 0, Change by more than one level = 1), × 6: field base points

*PHD* physical domain, *PSD* psychological domain, *SOD* social domain, *SSD* common symptoms and side effect domain, *CGM* core/general module, *SPD* specific domain, *TOT* total

## Discussions

In this study, anchor-based method, ROC curve method and the multiple linear regression models were used for both two standards, providing a basis for selecting the appropriate MCID.

The correlation coefficients between item Q29 and various domains are mostly higher than 0.30, showing a relatively strong correlation. Therefore, Q29 can be used as a subjective anchor.

The traditional anchor-based method is relatively simple. Because the sample size is large and the data is normally distributed, the mean value of the difference is taken, and the MCID value obtained is slightly larger than the other two methods. The anchor method has fewer restrictions. It can provide a professional explanation for the determined through the relationship with the effect standard. Generally, the user can choose this method to obtain the MCID value, which directly and simply reflects the change of the patient’s quality of life. If the sample size is small, the median of the difference can be chosen as MCID. The disadvantage of this method is that it does not consider measurement errors.

The ROC curve method combines an anchor-based method with a distribution-based method, the size of change can be described by sensitivity and specificity and also AUC curve. The area under the ROC curve shows the rationality of the selected anchors, the AUC of this study is basically above 0.7, indicating a good effects, the cut-off point is the Yorden Index. The MIC corresponding sensitivity and specificity are visualized, increasing the precision and accuracy of the MID estimation. The characteristic of ROC curve method is relatively stable. The MCID produced by the two standards in this study is almost the same. The ROC approach is suitable for data that is not normally distributed, and can use the entire data set, thus maximizing precision [27]. When evaluating the effectiveness of clinical interventions and requiring higher standards (higher sensitivity), this method is relatively suitable. It is difficult to identify the cut-off point with the best sensitivity and specificity at one glance in the ROC curve. Therefore, Terluin [28] introduced an alternative to the ROC-based MIC, based on predictive modeling.

Multiple linear regression models are used less frequently. *R*^*2*^ in this study was lower, possibly because some independent variables had little effect on dependent variables although the model was significant with all *P* values being less than 0.05. This method can effectively control the influence of confounding factors on MCID, and can identify the confounding factors that have the greatest influence on MCID [29]. For example, this study found that the patient's group (self-assessment change level) and family economic conditions have a greater impact on the quality of life scores in various domains. Therefore, this method can be chosen when need to find factors that affect postoperative recovery or clinically affect the patient's treatment effect, etc. But the sample size requires more and more as factors and their levers increasing.

The similarity between the multiple linear regression model and the ROC curve method is that the sample sizes of standard A and B are the same. The difference is that multiple linear regression models use basic patient information and can generate 95% confidence intervals to predict the individual mean and the population mean, while the ROC curve method can increase the precision and accuracy of the MCID estimation.

To sum up, in terms of the three methods, MCID value ranked as follows: anchor-based > ROC curve > multiple linear regression model, and the two standards of ROC curve method produced almost the same MCID.

In addition to the above methods, some other methods are gradually studied and used, such as Logistic regression model, response cumulative distribution function and so on. They can be also used to calculate MCID for the scale of QLICP-ES in the future.

Regarding the selection of effect size on MCID, some studies [30–32] have pointed out: it is critical that the MCID score is a valid and stable measure for clinicians and researchers alike. A low MCID value may result in overestimating the positive effects of treatment, but it may be appropriate for screening purposes, whereas a high MCID value may incorrectly classify patients as failing to respond to treatment when in fact the treatment was beneficial [30]. The median value may be a better estimate of true and meaningful change when applying a conservative standard for evaluating treatment effects or for respondent analyses [31, 32]. In our study, in terms of the two standards, the MCID obtained from standard B is slightly larger than that from standard A, but standard A "one grade difference" can reflect the minimum clinical difference directly.

It is wealthy to note the issue of sample size. After consulting the literature, we found that there is no focused discussion and formula calculation for the sample size of MCID. Therefore, the sample size of this study follows the empirical principle of evaluation of the scale (usually bigger than 100, i.e. thumb principle). The 157 and 220 cases in our study are the calculated number of patients included under the two standards, 157 patients under the A standard, and 220 patients under the B standard. Obviously, it needs study further in future on sample size of MCID.

To sum up, the different methods produce different MCID values. A variety of different MCID values and methods are given in the article, and users can refer to them according to the research purpose and sample characteristics. Despite all this, in consideration of compromise and stability, the recommended MCID values of physical domain, psychological domain, social domain, common symptom and side-effects domain, the specific domain and the overall were 7.8, 9.7, 4.7, 3.6, 4.3, 2.3 and 2.9, respectively. Obviously, it was calculated according to ROC curve method under standard A.

## Conclusions

Different methods have their own advantages and disadvantages, and also different definitions and standards can be adopted according to research purposes and methods. In this paper, a lot of different MCID values were presented and prompted the application scenario. It can be easy and convenient to select by users according to different situations, and also considering recommend results. The purpose of this study is to explore MCID formulation methods and provide the basis for selecting the appropriate MCID. As a hot and difficult problem, MCID needs to be studied further.

## Data Availability

Not applicable.
